# Sepsis Biomarkers in Evolution: Comparative Insights and the Promising Roles of MDW and Presepsin

**DOI:** 10.3390/medicina62010148

**Published:** 2026-01-12

**Authors:** Andrea Piccioni, Lucrezia Fiorentino, Silvia Baroni, Simone Leggeri, Giulia Pignataro, Giulia Napoli, Gabriele Savioli, Marcello Covino, Antonio Gasbarrini, Francesco Franceschi, Marcello Candelli

**Affiliations:** 1 Department of Emergency Medicine, Fondazione Policlinico Universitario Agostino Gemelli-IRCCS, 00168 Roma, Italy; 2 Faculty of Medicine, Università Cattolica del Sacro Cuore, 00168 Roma, Italy; lucrezia.fiorentino26@gmail.com (L.F.); silvia.baroni@unicatt.it (S.B.);; 3 Department of Laboratory and Infectious Sciences, Fondazione Policlinico Universitario Agostino Gemelli-IRCCS, 00168 Roma, Italygiulianapoli.93@libero.it (G.N.); 4 Departement of Emergency, IRCCS Fondazione Policlinico San Matteo, 27100 Pavia, Italy; 5 Medical and Surgical Science Department, Fondazione Policlinico Universitario Agostino Gemelli-IRCCS, 00168 Roma, Italy

**Keywords:** sepsis, biomarkes, MDW, presepsin, diagnosis

## Abstract

*Background and Objectives:* Sepsis is a life-threatening condition caused by a dysregulated host response to infection. Early recognition is crucial to improve outcomes, but conventional biomarkers such as C-reactive protein (CRP) and procalcitonin (PCT) show limited diagnostic accuracy. *Materials and Methods:* We performed a narrative review of the literature on sepsis biomarkers, with a focus on their biological role, diagnostic performance, clinical applicability, and limitations. Particular attention was given to presepsin (P-SEP) and monocyte distribution width (MDW), which have recently gained relevance. *Results:* Several novel biomarkers—including lipopolysaccharide-binding protein (LBP), soluble triggering receptor expressed on myeloid cells-1 (sTREM-1), mid-regional pro-adrenomedullin (MR-proADM), neutrophil gelatinase-associated lipocalin (NGAL), Proenkephalin (PENK), and circulating microRNAs—have been studied, though most remain investigational. Among them, P-SEP shows rapid kinetics and correlation with disease severity, while MDW, derived from routine complete blood count, offers encouraging sensitivity and cost-effectiveness in emergency settings. Both biomarkers appear practical and potentially valuable for early sepsis detection. *Conclusions:* P-SEP and MDW emerge as the most promising biomarkers for timely sepsis recognition and risk stratification. Further validation and standardization are required to include them into routine clinical practice.

## 1. Introduction

Sepsis is a life-threatening syndrome caused by a dysregulated host response to infection. According to the Sepsis-3 definition, sepsis is characterized by an acute increase of at least two points in the Sequential Organ Failure Assessment (**SOFA**) score in the context of suspected or confirmed infection [1].

Despite advances in supportive care, sepsis remains one of the major causes of morbidity and mortality worldwide [2]. Early recognition and timely antimicrobial therapy are crucial for improving patient outcomes, as delayed diagnosis significantly increases the risk of progression to severe sepsis and septic shock.

Recently, there has been growing interest in identifying reliable biomarkers that can facilitate early sepsis diagnosis, guide clinical decision-making, and improve prognostication.

This review aims to provide an overview of the prior biomarkers reported in the literature for sepsis diagnosis and to compare their characteristics, advantages, and limitations. A wide range of biomarkers that correlate with various aspects of the septic response, such as inflammation, endothelial dysfunction, immune activation, and cellular injury, has been analyzed. Among these, a key focus will be on monocyte distribution width (MDW) and presepsin (P-SEP), which are emerging as the most promising tools for early detection and risk stratification.

## 2. Methods

The literature search was performed using the following electronic databases: PubMed and Scopus. Additional relevant studies were identified through manual screening of the reference lists of key articles and recent reviews. The search included articles published from January 2000 to July 2025, reflecting both historical biomarkers and recently developed parameters.

Search terms included combinations of the following keywords: *“sepsis”*, *“biomarkers”*, *“diagnosis”*, *“prognosis”*, *“MDW”*, *“presepsin”*, *“procalcitonin”*, *“C-reactive protein”*, *“NGAL”*, *“sTREM-1”*, *“MR-proADM”*, “*proenkephalin*” *“lipopolysaccharide binding protein”*, and *“microRNA”*. Boolean operators (AND/OR) were applied to refine the search strategy.

Study were considered eligible if they met the following inclusion criteria:

1. Focus on sepsis or septic shock according to recognized diagnostic criteria;
2. Evaluation of one or more biomarkers for diagnosis, severity assessment, or prognosis;
3. Original research articles, meta-analyses, or high-quality narrative reviews;
4. Published in peer-reviewed journals and written in English.

Exclusion criteria were

1. Studies not directly relevant to the diagnostic or clinical utility of sepsis biomarkers;
2. Articles lacking original data or meaningful clinical insight.

A narrative review methodology was chosen instead of a systematic review due to the heterogeneity of study designs, patient populations, clinical settings (emergency department vs. intensive care unit), analytical methods, and outcome measures across the available literature. This approach allowed a broader and clinically oriented discussion of biomarker performance, limitations, and applicability.

## 3. Current State of Biomarkers

### 3.1. Most Studied Biomarkers: C-Reactive Protein (CRP) and Procalcitonin (PCT)

**C-reactive protein (CRP) and procalcitonin (PCT)** are the most widely used laboratory biomarkers for the diagnosis and monitoring of sepsis in clinical practice. CRP is an acute-phase protein synthesized by the liver in response to pro-inflammatory cytokines, particularly interleukin-6 (IL-6) [3,4]. Its main role in innate immunity is to bind to phosphocholine residues on the surface of pathogens (such as bacteria, parasites, and fungi) and damaged cells, activating the complement pathway and promoting opsonization. CRP levels start to rise within 6–8 h after the onset of inflammation, peaking at around 48 h [4,5]. Albeit highly sensitive to inflammation, CRP is non-specific, as it can be elevated in a wide range of infectious and non-infectious conditions, including trauma, surgery, and autoimmune disorders. In sepsis, reported sensitivity for CRP ranges from 72% to 92%, with specificity often below 70%, limiting its discriminatory value between infectious and non-infectious systemic inflammation [6,7].

PCT, the prohormone of calcitonin, is normally produced by thyroid C cells. In bacterial infections, however, PCT is synthesized by multiple tissues in response to bacterial endotoxins and pro-inflammatory cytokines such as IL-1β and TNF-α, independently of calcitonin regulation [8]. Viral infections typically induce interferon-γ production, which suppresses PCT release, contributing to its higher specificity for bacterial infections [9]. PCT levels begin to rise within 3–6 h of infection onset and peak at 12–24 h, making it an earlier marker than CRP [10]. The half-life of PCT is between 20 and 24 h; therefore, when a proper host immune response and antibiotic therapy are in place, PCT levels decrease by 50% over 24 h [11]. Multiple studies have reported sensitivities for sepsis ranging between 70% and 85% and specificities between 70% and 91%, with higher accuracy in differentiating bacterial causes of systemic inflammation from non-bacterial ones compared to CRP [12,13]. However, PCT levels could also be elevated in certain non-infectious conditions, such as major surgery, severe trauma, or prolonged cardiogenic shock, and might remain low in localized infections.

Overall, both CRP and PCT are useful for initial assessment and follow-up in suspected sepsis. However, CRP is highly sensitive but non-specific, and while PCT offers greater specificity and disease-monitoring utility, its sensitivity can be limited in very early sepsis.

### 3.2. MiRNA

Numerous research studies shed light on the role of miRNAs in the physiopathology of sepsis. MiRNAs are endogenous non-coding RNA molecules of approximately 21 nucleotides in length [14]. They play essential roles in immune responses and inflammation; dysregulated miRNA expression profiles have been identified during sepsis, thereby suggesting their potential utility as diagnostic and prognostic biomarkers [14].

In a pediatric cohort study [15], miR-122 and miR-146a were the most promising biomarkers identified: miR-122 showed an AUC of 1.00 with a cut-off value of 1.29, sensitivity of 97.5%, and specificity of 100%; similarly, miR-146a demonstrated an AUC of 1.00 with a cut-off of 0.73, reaching 100% sensitivity and specificity.

In a separate pilot study focused on T cell immunoparalysis during sepsis [16], a miRNA microarray was performed on RNA isolated from T cells of both septic patients and healthy controls. A total of 35 miRNAs were found to be differentially expressed; among them, the most dysregulated were miR-150, which was markedly downregulated (AUC 0.91; 95% CI: 0.80–1.00), and miR-143, which was upregulated (AUC 0.95; 95% CI: 0.88–1.00).

The main limitation of miRNAs in the diagnosis of sepsis is the complexity of the analytic method. It entails RNA isolation and reverse-transcription and quantitative PCR, requiring the samples to be stored at −80° C. In addition, miRNA signatures may vary with aging, meaning that analyzing miRNA differences should take into account age-matched cohorts for more accurate analyses [17].

Furthermore, **the reproducibility of findings is compromised by the lack of a standardized methodological approach**. For instance, **some studies analyze miRNA expression in serum**, while **others use plasma**, a variation that could influence the results. The inconsistency underlines the urgent need for protocol harmonization in future research in order to better evaluate the diagnostic performance of miRNAs in sepsis.

Overall, despite promising diagnostic accuracy in experimental and small clinical cohorts, the clinical applicability in emergency department settings of miRNAs remains limited because of their long turnaround time, high costs, and need for specialized laboratory infrastructure.

### 3.3. Soluble Triggering Receptor Expressed on Myeloid Cells-1 (sTREM-1)

TREM-1 (Triggering Receptor Expressed on Myeloid cells 1) is expressed on monocytes and neutrophils. During inflammation, the soluble form sTREM-1 is released in plasma to amplify the innate immune response.

Its potential use as an early-sepsis biomarker has been recently evaluated in different studies, especially to differentiate sepsis from SIRS conditions.

A meta-analysis [18] comprising eleven studies and a total of 1795 patients reported sensitivity and specificity below 80%, indicating limited reliability of sTREM-1 when used as a standalone marker for sepsis diagnosis.

Recent clinical studies [19] have identified a cut-off value >133 pg/mL with sensitivity and specificity around 70%, but with significant limitations due to small sample sizes and poor standardization.

Furthermore, a separate study [20] showed that although sTREM-1 levels were higher in patients with sepsis compared to healthy subjects, it did not effectively discriminate between sterile SIRS, infectious SIRS, and sepsis (SIRS plus infection vs. sepsis *p* = 0.871; SIRS plus infection vs. sterile SIRS *p* = 0.72; sepsis vs. sterile SIRS *p* = 0.65), thereby limiting its clinical relevance.

Despite initial enthusiasm, sTREM-1 has shown conflicting diagnostic results across studies, with moderate sensitivity and specificity. The lack of standardized cut-off values and assay methods, together with limited added value compared to established biomarkers, restricts its clinical application in early sepsis diagnosis.

### 3.4. Neutrophil Gelatinase-Associated Lipocalin (NGAL)

NGAL is produced by neutrophils and tubular kidney cells in response to damage. AKI (acute kidney injury) represents one of the earliest organ dysfunctions observed during sepsis and is reported in about two-thirds of patients with septic shock [21,22]. Therefore, it is reasonable to consider AKI as an early sign of sepsis.

Because NGAL reflects neutrophil activation, which is typical of sepsis, it could play an important role in the early diagnosis and stratification of septic patients. Several studies suggest that NGAL measurements may assist in recognizing septic patients and in assessing disease severity, supporting the interest in its clinical application [23].

In a study where 5599 randomly selected people were prospectively followed for 10 years, neutrophil leukocyte count was found to be the main determinant of plasma NGAL in the general population, with kidney function instead being less relevant [24].

Accordingly, an Indian prospective study [25] evaluated the diagnostic value of NGAL in sepsis, ignoring kidney damage. It showed that an increase of 100 mg/dL in plasma NGAL corresponded to a relative risk of bacterial sepsis of 1.04 (95% CI: 1.01–1.06). This association remained unchanged after adjustment for creatinine (despite its established role as a marker for AKI), implying that NGAL could be an independent marker of sepsis, using a cut-off value of 570 ng/mL. When plasma NGAL values were categorized according to this cut-off, individuals above the threshold exhibited a more than threefold increased crude risk of sepsis (RR ratio of 3.30, 95% CI: 1.41–7.72).

However, it is important to note that a lack of standardized assay methods and cut-off thresholds across studies limits the reproducibility and clinical application of NGAL as a sepsis biomarker. Future studies could better evaluate its role in sepsis.

In addition, it is not clear whether NGAL levels are influenced by obesity and insulin-resistance or not, with contrasting opinions in different studies [26,27].

### 3.5. Proenkephalin (PENK)

PENK is a 243-amino-acid opioid cleaved from the common endogenous opioid precursor. It interacts with delta-receptors for morphine, located in the central nervous system and kidney. It has been shown to improve renal function and to have cardio-depressive action through inhibition of catecholaminergic neural stimulation of the heart and direct negative inotropy [28].

In a prospective pilot study [29] including 23 patients admitted to ICU for sepsis or septic shock, PENK levels were shown to be more accurate than creatinine in evaluating kidney function in critically ill patients.

In another prospective study that aimed to compare PENK and NGAL, PENK was superior to NGAL in predicting AKI (*p* = 0.022) and renal replacement therapy (RRT) (*p* = 0.0085). Unlike NGAL, PENK was not influenced by inflammation and predicted 30-day mortality [30].

PENK appears to be a reliable biomarker to assess organ dysfunction and therefore to predict outcomes in septic patients. On the other hand, it has mainly had a role in predicting sepsis-related AKI rather than in diagnosing sepsis itself. Further studies could help clarify its potential integration into multimodal diagnostic panels.

### 3.6. Llipopolysaccharide-Binding Protein (LBP)

LBP is a plasmatic protein mainly produced by the liver which has a crucial role in recognition of LPS present on the outer membrane of Gram-negative bacteria. Once LBP binds LPS, the LPS/LBP complex is presented to CD14 to trigger monocyte activation, leading among other mechanisms to cytokine synthesis, necessary for the host response [31].

Notably, elevated levels of LBP have been observed not only in Gram-negative infections but also in **Gram-positive infections**. This is because LBP behaves as a non-specific marker of the systemic inflammatory response rather than a pathogen-specific signal [32,33]. Additionally, some evidence suggests that LBP can interact with other bacterial components such as lipoteichoic acid (LTA) from Gram-positive bacteria and inhibits the integration of LBP into phospholipid membranes, indicating the formation of complexes of LTA and soluble LBP [34].

Despite its role in triggering the innate immune response, its clinical performance as a sepsis diagnostic tool remains limited and controversial.

In a prospective cohort study [35], serum concentration of LBP was detected in patients with systemic inflammatory response syndrome (SIRS), sepsis, and septic shock to evaluate its ability to differentiate between infectious and non-infectious etiologies in SIRS and to predict prognosis. No statistically significant difference was found between patients with SIRS and those with sepsis (*p* = 0.61), suggesting that LBP is a **non**-**specific marker of acute-phase response** rather than of infection-specific inflammation.

A small prospective study measured LBP and other sepsis biomarkers, including PCT and CRP, on admission in 102 adult patients presenting with suspected infection. Considering a cut-off value of 20 μg/mL, ROC curve analysis and area under the curve (AUC) revealed a value of 0.701 for LBP, similar to CRP (0.707) and lower than that for PCT (0.844) (*p* = 0.012) [36]. The diagnostic accuracy of LBP for sepsis was similar to that of CRP but lower than that of PCT.

Another prospective study evaluating various biomarkers [37] reported that using a cut-off value of **20 μg/mL** for LBP resulted in a **sensitivity of 78.3%** and a **specificity of 64.2% in** distinguishing between patients with non-infectious SIRS and patients with sepsis/severe sepsis. When a higher cut-off value was applied (40 **μg/mL)**, **specificity increased significantly to 91%**, but this came at the cost of a marked reduction in **sensitivity to 37.7%.**

In conclusion, despite its involvement in the early immune response to bacterial infections, LBP has shown **limited effectiveness in accurately discriminating sepsis from non-infectious inflammatory conditions,** reducing its usefulness as an early diagnostic or rule-out biomarker. Overall, the available evidence suggests that LBP **lacks the diagnostic precision required for reliable standalone use** in sepsis detection and might be more appropriate as part of a **multi-marker approach** rather than as a single discriminative tool. The marked variability in reported cut-off values further highlights the heterogeneity of available evidence and limits its practical applicability in emergency settings.

### 3.7. Monocyte Distribution Width (MDW)

MDW measures the volumetric distribution width of monocytes. Monocytes rapidly respond to pathogen-associated molecular patterns (PAMPs) in the early stages of infection or systemic inflammation and undergo cytoskeletal remodeling and vacuolization, leading to increased heterogeneity in cell size. This variation is quantified by MDW, and since these changes appear **before significant cytokine release or organ dysfunction**, MDW rises earlier than traditional markers like CRP or PCT [38,39,40].

MDW is integrated into the routine complete blood count (CBC) and measured automatically using modern hematology analyzers such as the Beckman Coulter DxH series. This allows for results to be obtained within 15–30 min from sample collection, with MDW itself calculated in under 2 min once analysis begins [41,42]. Its inclusion in the standard CBC offers a low-cost and rapid tool for early sepsis screening without requiring additional sampling or specialized assays.

In a meta-analysis conducted by our research group [40], which included 31 studies and over 39,000 patients, MDW showed a **sensitivity ranging from 75% to 87%** and a **specificity of approximately 70%,** depending on the cut-off applied (typically between 19.8 and 21.5). In addition, studies consistently reported that MDW correlates with disease severity and displays good overall diagnostic performance, with AUC values around **0.79–0.82**, often outperforming traditional biomarkers such as CRP and PCT.

A recent meta-analysis comprising 25 observational studies and 39,041 patients [43] reported a pooled sensitivity of 0.79 (95% CI, 0.74–0.83) and a specificity of 0.7 (95% CI, 0.61–0.78) for sepsis-2. When applying Sepsis-3 criteria, sensitivity was 0.83 (95% CI, 0.78–0.88) and specificity 0.64 (95% CI, 0.55–0.71).

One of the main limitations of MDW is the lack of a standardized cut-off, which may vary depending on the clinical context (ED vs. ICU) and in samples treated with different anticoagulants (K2EDTA vs. K3EDTA) [40].

Furthermore, the research shows low specificity of MDW in diagnosing sepsis, which may lead to false positives.

### 3.8. Presepsin (P-SEP)

P-SEP is a 13 kDa N-terminal fragment of the soluble form of CD14 (sCD14), generated through proteolytic cleavage by cathepsin D in plasma [44]. CD14 works as a co-receptor within the Toll-like receptor 4 (TLR4)/MD-2 complex. It is involved in the innate immune recognition of bacterial ligands, including lipopolysaccharide (LPS) from Gram-negative bacteria and peptidoglycans from Gram-positive bacteria [45,46].

Because P-SEP is released into the bloodstream following monocyte/macrophage activation, it is considered a promising early biomarker of sepsis. P-SEP rises **very early** after the onset of infection; detectable increases can occur **within 2–3 h** after bacterial stimulation, often preceding PCT and CRP [47]. This makes it particularly useful in emergency and critical care settings, where early identification of sepsis is crucial.

The need for rapid measurement has led to the development of the **PATHFAST^®^ system** (Mitsubishi Chemical), which uses chemiluminescence enzyme immunoassay (CLEIA) coupled with MAGTRATION^®^ technology. This allows quantitative measurement of P-SEP in whole blood in approximately **17 min**, processing up to six samples simultaneously, with a detection range of 0.05–3.00 ng/mL [48,49,50]. This is a significant improvement over previous ELISA-based methods, which required up to 4 h and had narrower analytical ranges.

Analyzing its diagnostic performance, several studies have demonstrated robust sensitivity and specificity. A meta-analysis by Zhang et al. (2015) reported a pooled **sensitivity of 84%** and **a specificity of 76%** for sepsis diagnosis [51]. More recently, a multicenter prospective trial using a cut-off of **600 pg/mL** showed a **sensitivity of 87.8%** and a **specificity of 81.4%** in differentiating sepsis from non-infectious conditions [52,53,54]. Another prospective study by Shozushima et al. (2011) found that P-SEP reached a diagnostic performance comparable or superior to that of procalcitonin (PCT), particularly in early sepsis, with an **AUC of 0.87, sensitivity of 80%,** and **specificity of 81%** at a cut-off of 647 pg/mL [55].

Our research group conducted a prospective study involving 216 patients [56] to compare the diagnostic performance of P-SEP and PCT. P-SEP demonstrated superior sensitivity (91.9% vs. 68.6%) and overall diagnostic accuracy (AUC 0.946 vs. 0.905). In addition, the kinetics of the two biomarkers were evaluated, showing that they may offer complementary insights: a decline in P-SEP with persistently elevated PCT could indicate ongoing systemic inflammation, supporting the need for continued antimicrobial therapy, close monitoring, and the use of serial measurements to guide treatment adjustment and de-escalation strategies.

Despite its promising performance, P-SEP has several limitations. Its levels can be influenced by **renal dysfunction**, leading to potential false positives in patients with chronic kidney disease or acute kidney injury [53,57,58]. Recent evidence suggests that different cut-off values should be applied according to renal function, as hypercreatinemia significantly affects P-SEP concentrations while preserving its prognostic value for mortality [58].

Furthermore, the **cost and limited availability** of CLEIA-based analyzers in some settings may restrict its routine use.

### 3.9. MR-Proadrenomedullin (MR-proADM)

MR-proADM is a fragment of 48 amino acids that splits from the proAdrenomedullin (proADM) molecule in a 1:1 ratio with Adrenomedullin (ADM) and therefore proportionally represents its levels and activity [59]. Unlike ADM, MR-proADM is stable in blood, making it a more reliable biomarker.

ADM is produced by a wide range of cells, including vascular endothelial and smooth muscle cells, especially upon stimulation with inflammatory cytokines [60], and plays a part in vasodilator, positive inotropic, diuretic, natriuretic, and bronchodilator functions [61], being therefore involved in clinical manifestations of sepsis. For instance, levels of MR-proADM may reflect the severity of organ dysfunction, even in the first stages of the disease, as well as track the escalation of systemic inflammation, the evolution from sepsis to septic shock, and the mortality risk of septic patients [62].

In a prospective study [63], PCT and MR-proADM were evaluated along with the SOFA score to analyze their diagnostic accuracy individually and combined. An MR-proADM cut-off of at least 1.50 nmol/L showed an AUC of 0.81 (CI 95% 0.73–0.88) in patients with sepsis. When patients were stratified into two groups—those with a confirmed microbiological isolate and those without—sensitivity and specificity were 87% and 68% in the group with an isolate and 74% and 78%, respectively, in the group without. To diagnose sepsis, the best combination was PCT with MR-proADM, with a post-test probability of 0.988. 

A meta-analysis reviewed 40 different studies that compared the diagnostic performance of P-SEP and MR-proADM in sepsis [64], concluding that the sensitivity of P-SEP was higher than that of MR-proADM (0.86 vs. 0.84, *p* < 0.001), whereas the specificity of MR-proADM was higher than that of P-SEP (0.79 vs. 0.86, *p* < 0.001). The cut-off value range was 89.26–1315 pg/mL for P-SEP and 0.8–1.165 pg/mL for MR-proADM.

Overall, while MR-proADM demonstrates promising diagnostic accuracy when combined with other biomarkers such as procalcitonin, the current evidence highlights its predominant value as a prognostic indicator of disease severity, organ dysfunction, and mortality risk in septic patients, rather than as a standalone tool for the initial diagnosis of sepsis.

## 4. Comparison

### 4.1. Publication Trends

Over the years, a significant increase in the number of scientific publications focused on emerging biomarkers in sepsis has been observed, reflecting the growing interest in enhancing early diagnosis and risk stratification of this complex syndrome.

**Figure 1:**
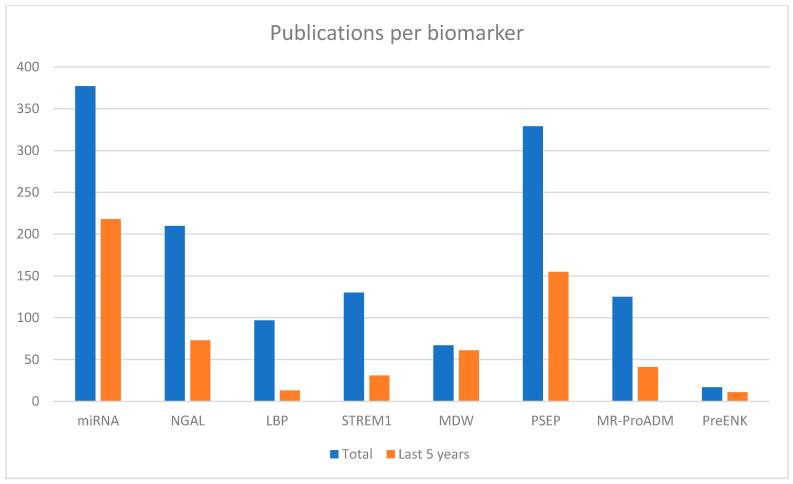
Number of publications retrieved from PubMed for each biomarker using the search terms “[biomarker name] AND sepsis diagnosis.” The search was performed on 17 June 2025. Only articles published in English were included, with no restrictions on study type. For each biomarker, the total number of publications is shown alongside the number of publications from the last five years.

A quantitative bibliometric analysis based only on PubMed publications reveals distinct trends in scientific attention towards these biomarkers, as shown in Figure 1.. MiRNAs have attracted a large volume of publications, partly due to their vast diversity and multiple types, which contribute to the high number of studies. While established markers like NGAL, sTREM-1, and MR-proADM continue to receive steady attention, their recent publication rates are proportionally lower. Lipopolysaccharide-binding protein (LBP) shows a declining trend. Proenkephalin, despite being less extensively studied, has had the majority of publications in the last five years.

Notably, biomarkers such as MDW and P-SEP exhibit marked surges in recent scientific output, indicating emerging prominence in sepsis research. MDW has seen 91% of studies published in the last five years, underscoring its rapid emergence as a potentially valuable biomarker. Although P-SEP has been extensively investigated, it continues to attract significant research interest.

### 4.2. Comparative Table

Several analytical and clinical factors influence the applicability of biomarkers in sepsis diagnosis and risk stratification. In particular, turnaround time, cost, and diagnostic performance are critical for their integration into routine clinical practice. A summary of these characteristics for the most commonly investigated biomarkers is provided in Table 1.

### 4.3. Different Expression Profiles of Biomarkers Based on the Infectious Trigger

Biomarkers display different expression profiles depending on the infectious trigger, as shown in Table 2.

PCT is mainly associated with bacterial infections, with typically higher levels in Gram-negative compared with Gram-positive sepsis. Viral infections, on the other hand, suppress PCT via interferon-γ activity. In Candida infections, PCT levels may rise modestly, particularly when there is an associated bacterial coinfection [12,77]. CRP is a sensitive but non-specific inflammation biomarker, as it increases in almost all infectious settings, including bacterial, viral, and fungal disease [78]. LBP typically increases in Gram-negative infections; recent studies showed moderate increases in Gram-positive and in fungal sepsis [79]. sTREM-1 is consistently induced in both Gram-negative and Gram-positive sepsis and has also been studied in invasive candidiasis, where increased levels have been documented in experimental settings [80]. NGAL has mainly been investigated in bacterial Gram- infections, particularly in relation to kidney injury, whereas data on viral and fungal contexts are still scarce. MDW, in contrast, rises across bacterial, viral, and fungal sepsis, reflecting morphological changes in monocytes that accompany systemic inflammation, regardless of the pathogen involved [43,81]. P-SEP has been widely validated in bacterial sepsis, but it also shows promising performance in candidemia, with values correlating with severity. Some studies have also suggested increases in viral infections such as influenza and COVID-19, although the evidence is more limited [82,83]. Finally, microRNAs (miRNAs) are deregulated in sepsis of bacterial, viral, and fungal origin, and although still experimental, they represent an exciting field for the development of novel diagnostic and prognostic tools [84,85].

## 5. MDW and P-SEP: Overview

### 5.1. Focus on MDW and P-SEP

MDW and P-SEP have emerged as promising tools in the context of sepsis as they could be complementarily used for early detection and patient monitoring. MDW offers significant advantages in routine clinical workflows, as it does not require any additional sampling, has a very short turnaround time (approximately 15–30 min), and incurs no extra cost. **MDW could be incorporated into routine hematological testing even in cases where sepsis is not the primary diagnostic suspicion**, facilitating early identification of evolving infection in patients presenting with non-specific clinical signs. On the other hand, P-SEP provides valuable information for both early diagnosis and dynamic assessment of disease progression. Its kinetics are favorable, with concentrations rising within 2–3 h of the infectious trigger—substantially earlier than CRP and comparable to or faster than PCT. P-SEP demonstrates high sensitivity (80–87%) and specificity (67–81%) and can be measured rapidly via point-of-care or ELISA-based assays within 15–30 min. Notably, **persistent or increasing P-SEP levels over time have been associated with worse clinical outcomes**, suggesting that serial measurement may be useful for monitoring response to therapy and identifying patients who may require escalation of care [69].

### 5.2. Ongoing Project

A prospective study is currently being conducted in our Emergency Department, focusing on the early diagnosis of sepsis. Patients with suspected sepsis are being enrolled, and both P-SEP and MDW are being analyzed to assess their individual and combined potential in improving early diagnostic accuracy. Results from this study will be reported in future publications.

## 6. Conclusions

Early diagnosis of sepsis still remains a clinical challenge, and conventional biomarkers such as CRP and PCT, while widely used, show limitations in sensitivity and specificity. Several biomarkers are under investigation—including LBP, STREM-1, MR-proADM, NGAL, and miRNAs—but P-SEP and MDW emerge as the most practical and clinically valuable at present. P-SEP has shown strong diagnostic and prognostic performance, with rapid kinetics and good correlation with disease severity. MDW is a parameter easily obtainable from routine complete blood count and has demonstrated encouraging sensitivity and specificity in emergency settings. Its main strength is being a cost-effective and readily available option. Future research should concentrate on the validation and standardization of these markers, as well as their integration with clinical scoring systems, to obtain earlier and more accurate sepsis detection.

## Figures and Tables

**Table 1:** Summary of biomarker characteristics, including turnaround time, estimated cost, sensitivity, specificity, and typical time of increase after sepsis onset. Sensitivity, specificity, and time of increase data were extracted from PubMed and Scopus searches using “[biomarker name] AND sepsis diagnosis” (search performed on 1 June 2025). Turnaround time and estimated cost were collected from additional studies and manufacturer information, independent of sepsis-specific studies. Only English-language sources were included; values are approximate and may vary between studies and settings.

Biomarker	Turnaround Time	Estimated Cost	Sensitivity	Specificity	Time of Increase	Main References
**CRP**	30–60 min	Very low (EUR 1–5)	72–92%	40–70%	Increases after 12–24 h	[4,6,65,66]
**PCT**	20–60 min (automated)	Medium (EUR 15–50)	70–85%	70–91%	Starts to rise at 6 h, peaks at 12–24 h	[12,13,66,67]
**miRNA (e.g., miR-146a, miR-150)**	6–24 h (RT-PCR)	High (EUR 100–200)	Variable (60–90%)	Variable	Very early, transcriptional response	[14,15,16,68]
**MDW**	~15–30 min (part of CBC)	Low (included in CBC)	75–85%	65–80%	Very early, during monocyte activation	[38,39,40,69]
**P-SEP**	15–30 min (POC or ELISA)	Medium (EUR 20–60/test)	80–86%	67–81%	Early, within 2–3 h from infectious trigger	[49,53,56,57]
**NGAL**	30 min–2 h (plasma)	Medium (EUR 20–60)	87% (cut-off 570 ng/mL)	46%	Early (2–4 h), but influenced by AKI	[24,25]
**LBP**	1–3 h (ELISA)	Medium (EUR 30–60)	~78%	~64%	Within 24 h (not specific)	[33,35,36,70,71,72]
**sTREM-1**	1–3 h (ELISA)	Medium (EUR 30–70)	71%	73%	Early, amplifies inflammatory signaling	[19,73]
**MR-proADM**	30–60 min	Medium (EUR 30–70)	81–84%	68–86%	Early, within 4–6 h	[74,75]
**Pre-ENK**	30–60 min	Medium (EUR 30–70)	N/A *	N/A *	Within 6–12 h from renal insult or systemic stress	[76]

* Data mainly available for AKI; direct role in sepsis diagnosis not yet established.

**Table 2:** Association of sepsis biomarkers with different types of infections: ✓ indicates positive association; ✗ indicates negative association.

Biomarker	Bacterial Gram−	Bacterial Gram+	Viral	Fungal
**PCT**	✓	✓ (lower)	✗/rare	✗
**CRP**	✓	✓	✓	✓
**LBP**	✓	✓ (less)	✗	✓ (some cases)
**STREM-1**	✓	✓	✗	✓ (some animal studies)
**NGAL**	✓	✗	✗	✗
**MDW**	✓	✓	✓	✓
**PSEP**	✓	✓	✓	✓ (mainly Candida)
**miRNA**	✓	✓	✓	✓

## Data Availability

The articles cited in this paper are available on PubMed^®^, UptoDate^®^ (Version 23.2) and Cochrane^®^ (Cochrane Database of Systematic Reviews, Issue 6, 2025).
